# Editorial: Improving the Way Science is Done, Evaluated, and Published

**DOI:** 10.1523/ENEURO.0373-17.2017

**Published:** 2017-11-06

**Authors:** Christophe Bernard

Dear friends and colleagues,

We are now three years old, and still growing! We recently published our 500th article, doubling the 2016 score, and the number of submissions is on the rise as compared to last year. Thank you, fellow neuroscientists, for choosing *eNeuro* to publish your research. What impresses me most is the quality of the science that *eNeuro* publishes. Many papers made it to the news this year, both in top scientific magazines and in the general media.

*eNeuro* has found its mark in the publishing landscape. The readership has more than doubled in a year. Given the plethora of scientific journals, it is impossible to read all the tables of contents. Most of us focus on the journals we trust, and where we know we will find the most relevant and reliable scientific information. It is particularly gratifying to see that *eNeuro* is such a journal. Both authors and readers make our success, so, thank you, all of you.

Note that our audience will continue to increase. We have teamed up with TrendMD, which recommends articles to readers based on what the person is reading at the moment, what they have read in the past, and the behavior of similar readers across the network. And this works well to draw in new readers as our journal articles are recommended in other journals with related articles. In just 6 weeks, TrendMD has helped increase traffic to *eNeuro*’s articles with an additional 1,500 article views from over a 1,000 new visitors. Thus, your papers will have a greater exposure.

As a society journal, we are here to serve the community. This is why, in addition to publishing classical research papers, we encourage the publication of papers in other categories such as “negative studies,” “failure to reproduce,” and “confirmation.” Surprisingly, we receive a comparatively low number of these. Perhaps, this is due to a lack of awareness that such categories exist. So, do not hesitate to send such papers; they are as important to the community as the more conventional ones.

As you now know, *eNeuro* actively participates in reshaping the publishing world, for the better. Did you notice that at least one commercial publisher of the main scientific generalist journals is experimenting with what *eNeuro* started: double blind review + consultation + synthesis? With other noncommercial journals, like *eLife*, we are leading the way, and we are constantly striving to innovate to improve the whole system.

**Let’s have a look at the past year’s achievements.**

**Enhancing scientific rigor**. We have adopted a new policy regarding modeling/theoretical papers, which now must include the computer code. We also strongly encourage including codes for in-house analysis software. This is not compulsory yet, but it makes sense to impose it. One step at a time. We may think that this is a constraint. But this is the only way to insure transparency and rigor.

**Extended data**. Extended data can be linked to figures and tables of the paper. They can take several forms, including large datasets. Extended data are not supplementary figures, they must convey an important message, without disrupting the logical flow of the paper. I think that it is an important addition.

**Improving the reviewing process**. We are both authors and reviewers. Yet, as authors, we always complain about the review process, in particular unnecessary experiments, unfairness, etc. The review system of *eNeuro*, consultation and synthesis, constitutes one way to solve these issues. A complementary approach is via a proper education of future reviewers. This year, we have released the 2nd webinar for Training reviewers, focusing on modeling papers. Both webinars are freely available for viewing. More webinars will come in the future.

**Acknowledging our work as reviewers**. We get more and more pressure to review papers, grants, etc. Such work is an integral part of Science, and we always feel that we are not appropriately acknowledged about it. This is the reason why whenever you perform a review for SfN journals (and others), it is integrated in your ORCID account. This information can then be used to assess objectively your reviewing work by committees for recruitment, promotion, etc.

## What’s next?

**Evaluating the evaluator**. At *eNeuro*, we are very proud of the system we set up (double blind and synthesis of reviews). We constantly receive positive, unsolicited, feedback from authors and reviewers. But it does not mean that we are perfect. Far from it. After all, this is a human endeavor. We accept, as authors, to be evaluated by a journal. But the reverse should exist. Hence, *eNeuro* will offer authors the possibility to evaluate our peer review process! Why? Because we need your feedback to constantly improve. I believe that it will highly contribute to the transparency of the review process. Stay tuned for that.

**Registered reports**. Several scientific journals offer the possibility to register a study before doing experiments. The study is evaluated for its potential scientific interest, and for the validity of the experimental protocol. This nearly guarantees publication, whatever the outcome of the experiments (i.e., if they are negative). We will soon implement registered reports.

**Communication**. We are hiring a features editor to help expand content. This will provide better exposure to the work you publish in *eNeuro*. We are also developing a blog page that will provide a place for communication about science between me and my editorial board, and you, the readers. As I mentioned above, this is a human endeavor, with much room for improvement. I rely on your help, suggestions, and feedback.

As you can see, we are doing well and we are still full of ideas to improve the way Science is done, evaluated, and published. But always remember: we are here to serve you and Neuroscience. This is our only purpose. Never hesitate to communicate with us at eNeuro@sfn.org.

Cheers,

